# Measuring Implementation Strength for Integrated Community Case Management in Malawi: Results from a National Cell Phone Census

**DOI:** 10.4269/ajtmh.14-0797

**Published:** 2015-10-07

**Authors:** Rebecca Heidkamp, Elizabeth Hazel, Humphreys Nsona, Tiope Mleme, Andrew Jamali, Jennifer Bryce

**Affiliations:** Johns Hopkins Bloomberg School of Public Health Baltimore, Maryland; Community Health Sciences Unit, Ministry of Health, Lilongwe, Malawi; National Statistics Office, Zomba, Malawi

## Abstract

Program managers, investors, and evaluators need real-time information on how program strategies are being scaled up and implemented. Integrated Community Case Management (iCCM) of childhood illnesses is a strategy for increasing access to diagnosis and treatment of malaria, pneumonia, and diarrhea through community-based health workers. We collected real-time data on iCCM implementation strength through cell phone interviews with community-based health workers in Malawi and calculated indicators of implementation strength and utilization at district level using consensus definitions from the Ministry of Health (MOH) and iCCM partners. All of the iCCM implementation strength indicators varied widely within and across districts. Results show that Malawi has made substantial progress in the scale-up of iCCM since the 2008 program launch. However, there are wide differences in iCCM implementation strength by district. Districts that performed well according to the survey measures demonstrate that MOH implementation strength targets are achievable with the right combination of supportive structures. Using the survey results, specific districts can now be targeted with additional support.

## Introduction

One challenge to program evaluation at scale is determining the strength of implementation, defined here as the quantity of a program delivered to a population. Too often, large-scale evaluations are not able to address questions of program effectiveness, because implementation is incomplete or of poor quality.^1^,^2^ Program managers face an equally important challenge in trying to assess and improve their programs without real-time information on the status of implementation, especially when expanding pilot or demonstration programs to scale. The quality of data produced by Health Management Information Systems—even when these systems include relevant data points—is often unknown and records are universally suspected to be incomplete.^3^ New and complementary methods are needed to produce up-to-the-moment snapshots of implementation strength for use in improving health service provision, calibrating routine monitoring systems, and interpreting changes (or lack of change) in measures of program outcome and impact.

Integrated Community Case Management (iCCM) is a community-based strategy that uses trained and supervised community health workers (CHWs) to assess, classify, and treat diarrhea, malaria, and pneumonia among children under 5 years of age.^4^ iCCM holds promise as a strategy to improve access to correct case management for the major infectious causes of child deaths.^5^ There is a growing body of implementation research addressing the challenges of scaling-up iCCM in low-income countries, and the first full evaluations of the strategy are starting to appear. The program assumption is that deploying iCCM-trained CHWs who are appropriately trained, supplied, and supervised, and at sufficient density in populations without access to fixed health facilities will improve access to appropriate treatment, will be used by the population, and will reduce child mortality from childhood pneumonia, diarrhea, and malaria.

Malawi was the first country in sub-Saharan Africa to implement iCCM at national scale.^6^ Beginning in 2008, the Ministry of Health (MOH), with support from the World Health Organization (WHO) and the United Nations Children's Fund (UNICEF), trained existing and newly recruited CHWs, who are called Health Surveillance Assistants (HSAs) in Malawi, and deployed them across 10 districts to areas that District Health Management Teams (DHMTs) defined as *hard-to-reach*, with limited access to fixed heath facilities. By 2010, the MOH was implementing the iCCM strategy in all 28 districts of Malawi, with support from various other donors and implementation partners (Box 1

Box 1 **iCCM in Malawi**

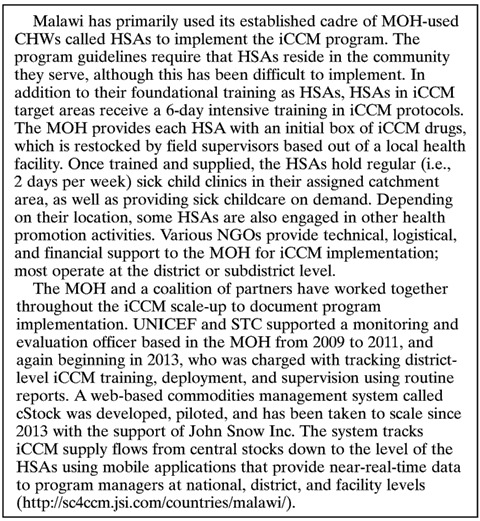

).

Starting in 2008, an independent evaluation team co-led by the National Statistical Office (NSO) and the Johns Hopkins Institute for International Programs worked with the MOH and WHO to document iCCM and other maternal newborn and child health programs in 10 districts. In 2011, an iCCM stakeholder technical working group (TWG) led by the MOH developed a list of consensus indicators for monitoring the strength of routine iCCM implementation at the level of the HSA and agreed to report on them regularly across all districts. In 2012, despite these efforts, the information on iCCM implementation remained incomplete. Therefore, the evaluation team collaborated with the MOH to develop, test, and implement a method to generate rapid, cross-sectional data on iCCM implementation through a cell phone survey.

We report here on the first full application of this phone-based implementation “snapshot” approach at national scale, conducted among a census of iCCM-trained HSAs in Malawi. A report on the validation study and cost of the method was published previously.^3^

## Materials and Methods

We conducted telephone interviews with all HSAs identified by the MOH as trained in iCCM and deployed to provide iCCM services. Enumeration of iCCM-trained HSAs started in May 2013. Interviews were completed between June and August 2013. Representatives from the Malawi MOH and implementing partners including WHO, UNICEF, and Save the Children (STC) reviewed and approved the study protocol. The Malawi National Health Sciences Research Committee and the Institutional Review Board at the Johns Hopkins Bloomberg School of Public Health provided ethical approval.

### Implementation strength indicators.

We selected the indicators to be measured from among those defined by the MOH and other iCCM stakeholders in 2011 (see Table 1 for a list of indicators and their definitions). We defined *currently working in iCCM* as an HSA who had seen at least one sick child in the 3 months before the survey. We included the small number of HSAs who completed on-the-job training rather than the official 6-day iCCM training course provided they met the currently working criterion. Each DHMT defines the *hard-to-reach areas* (HTRA) in their district using criteria established by the MOH. At time of the survey, these included areas more than 8 km from a health facility. However, there was no official listing available to match HSA catchment areas with these DHMT-defined HTRAs as is required to calculate TWG indicator 2a. Therefore, we asked HSAs to self-report whether they worked in HTRA and reported on this modified indicator. For indicator 3a, HSAs only reported the duration of the most recent stockout in the previous 3–6 months.

For indicators 4 and 5, *supervision* specifically refers a trained supervisor going out to the HSA's village clinic. iCCM-trained HSAs can also be *mentored* by qualified clinical staff when they come into the health facility. *Reinforcement of clinical practice* is defined as the supervisor either directly observing case management practices in the field or clinic or presenting case scenarios to the HSA. More general supervision activities such as protocol reviews or supply replenishing are not considered reinforcement of clinical practice. To distinguish supervision episodes that included reinforcement of clinical practice, HSAs were asked about the specific activities that occurred during their most recent supervision and mentorship experiences in the previous 3 months.

### Enumeration of HSAs.

We used a multistep process to identify all currently working HSAs who received iCCM training. District-level MOH officials provided lists of iCCM-trained HSAs organized by health facility. The study team called the in-charge of each health facility to review the accuracy and completeness of these lists. We used several approaches to further check the completeness of the enumeration process. In 22 of the 29 districts, we compared the lists to training and reporting records provided by nongovernmental organizations (NGOs) working in the area. In the seven districts without NGO data, we randomly selected 1–2 health facilities that district officials identified as not having iCCM activities and called the in-charge to verify whether this was actually true.

### Telephone interviews.

Teams from NSO and Johns Hopkins piloted the telephone interview guide in 2011, and in 2012, we conducted a validation study among 200 HSAs in Ntcheu and Dowa districts in Malawi.^3^ The final interview guide for the national census was translated into Chichewa from the original English, and the translation was field tested and back translated. Five teams of four interviewers and one supervisor each conducted all telephone interviews from a private room at the NSO headquarters in Zomba, Malawi. National MOH officials notified the DHMTs before the start of any interviews in their districts.

We attempted to contact each enumerated iCCM-trained HSA by cell phone. The interviewers followed a structured protocol that required up to eight documented call attempts over multiple days before an HSA was determined to be unreachable. If the interviewer was not successful in reaching the HSA within the first four call attempts over a 2-day period, he or she referred the case to the team supervisor who contacted the HSA supervisor or health facility in-charge to develop a strategy for reaching that HSA (e.g., arranging a time to call the HSA via the supervisor's or another HSA's phone). Verbal consent was obtained from each HSA before beginning the interview.

Supervisors were on site with the interviewers during data collection. They directly observed 5% of telephone interviews using a checklist to assess interviewer performance and provide individualized feedback. For quality control purposes, during the first 2 weeks of data collection, two questionnaires per week were randomly selected for each interviewer and the HSAs were called back and reinterviewed by the team supervisor.

### Other data collection.

The district-level MOH provided the total number of HSAs working in each district. The total district population under 5 years of age was taken from 2013 projections released by the NSO based on the 2008 National Census.^7^ We report on a total of 29 districts, because in 2009, the MOH divided Mzimba District into two management units, Mzimba North and Mzimba South.

### Data processing and analysis.

Responses captured on the paper questionnaires were double entered in CSPro (United States Census Bureau) and exported into STATA 11.0 (StataCorp LP, College Station, TX) for analysis.^8^,^9^ We calculated the proportion of all HSAs trained in iCCM by dividing the number of surveyed HSAs in each district who reported receiving the MOH's 6-day training by the total number of HSAs in each district as reported by district-level MOH officials. To facilitate interpretation across the 29 districts, we ranked and divided the districts into tertiles for select implementation strength and utilization indicators.

## Results

Figure 1

**Figure 1.**
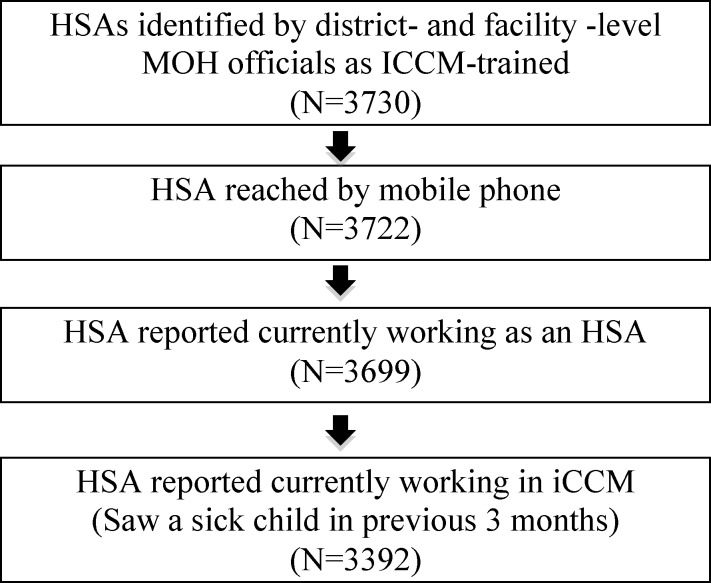
Overview of steps in identifying Health Surveillance Assistants (HSAs) currently working in Integrated Community Case Management (iCCM).

outlines the process of identifying HSAs currently working in iCCM. There were total 9,555 HSAs working in Malawi at the time of the survey. District-level MOH and facility in-charges identified 3,725 HSAs as trained in iCCM. The survey team reached all but eight of these HSAs by phone (99.7%). One HSA did not have a personal cell phone and was contacted using a supervisor's phone. Among those reached, 3,392 (91.4%) reported having seen a sick child in the previous 3 months and were classified as currently working in iCCM for the analysis.

Table 2 presents results for TWG indicators 1 and 2a. The mean density of deployed HSAs per 1,000 under 5 children was 3.3. The highest density was in Likoma, a sparsely populated island in Lake Malawi, and the lowest density was in predominantly urban Lilongwe District. Nationally, 38.4% of HSAs were trained in iCCM. District-level results ranged from 10.3% trained in Mangochi and 11.7% in Lilongwe compared with over 90% in Likoma, Mwanza, and Neno.

Table 3 provides an overview of demographic characteristics and work history of the interviewed HSAs. The majority were male (72.3%) and in their mid-30s. Almost all had at least some secondary education (97.2%), which is consistent with requirements for the Malawi Civil Service. The survey participants had been working as HSAs for an average of about 9.5 years including 2.5 years of iCCM experience.

**Figure 2.**
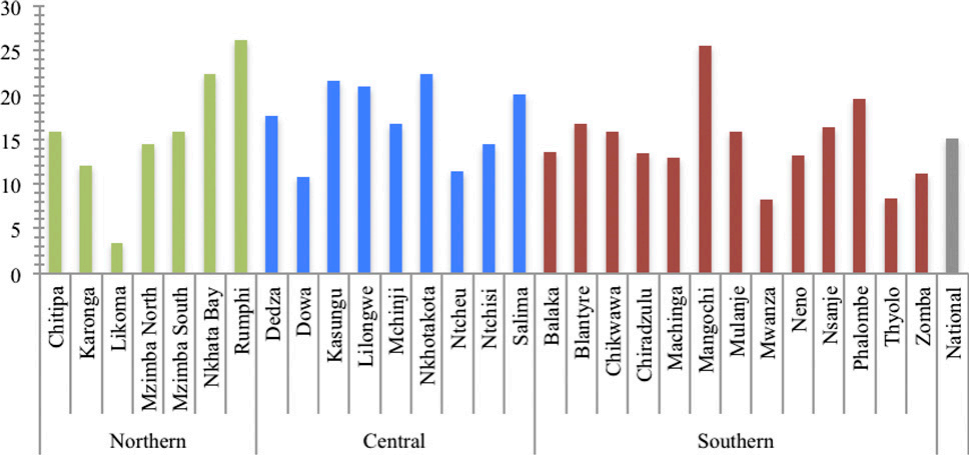
Mean number of sick children treated in the previous 7 days by district among Health Surveillance Assistants (HSAs) currently working in Integrated Community Case Management (iCCM) who had seen at least one child in the previous 7 days.

Table 4 presents the findings for the remaining TWG indicators. Consistent with the national strategy for iCCM targeting, the majority (87.1%) of surveyed HSAs self-reported working in a HTRA including three districts—Likoma, Blantyre, and Chiradzulu—where all surveyed HSAs reported working in HTRA. Regarding deployment indicator 2c, 77% percent of HSAs currently working in iCCM reported treating a sick child in the previous 7 days. These HSAs saw an average of 15 children in the previous week (Figure 2

). The actual number of sick children treated by each HSA varied widely both within and between the districts (Supplemental Table 1).

According to MOH strategy, HSAs are supposed to live in the catchment areas where they provide services (Box 1). TWG indicator 6 measures compliance with this policy. Nationally, 69.5% of HSAs working in iCCM reported living in their catchment area. The island of Likoma was the outlier, with only one of its six HSAs currently working in iCCM living in his or her catchment area.

Nationally, nearly 60% of HSAs reported no stockouts longer than 7 days for the four primary iCCM drugs. Five districts—Likoma, Mzimba North, Nkhata Bay, Rumphi, and Nsanje—met the 80% target set by the MOH for indicator 3a. The lowest rates were in Mchinji, Mwanza, and Mangochi. Districts varied in which individual drugs were more likely to be stocked out. HSAs were much less likely to be stocked out of any single iCCM drug compared with paracetamol and eye ointment, which are included in HSA drug kits, but not used for iCCM protocols (results not shown).

Lumefantrine–artemether (LA), co-trimoxizole, and oral rehydration solution are essential for timely life-saving treatment of malaria, pneumonia, and diarrhea, respectively. Therefore, a stockout of any of these drugs is considered unacceptable under TWG indicator 3b. Nationally, half of HSAs reported no stockout of life-saving drugs in the previous 3 months. Only three districts met the 80% MOH target. The lowest rates were in Mchinji and Mwanza, where only 20% of HSAs reported no stockouts of any life-saving drugs in the previous 3 months.

Indicators 4 and 5 relate to supervision of HSAs carrying out iCCM activities. Nationally, 43.7% of iCCM HSAs received field-based supervision and 42.0% received clinic-based mentorship in the previous 3 months. Over half of HSAs (57.6%) reported receiving field-based supervision and/or mentorship with reinforcement of clinical practice in the previous 3 months. Supervision and/or mentorship rates with reinforcement of clinical practice varied by district, with a high of 96.0% in Kasungu and a low of 21.7% in Chitipa. In some districts (e.g., Salima and Mulanje), rates of facility-based mentorship were higher than field-based supervision, while in others (e.g., Chitipa and Kasungu), field-based supervision was more common than facility-based mentorship (results not shown).

## Discussion

This is the first report of a cross-sectional implementation strength snapshot for iCCM. The results show that Malawi has made substantial progress in the scale-up of iCCM service provision since the launch of the program in 2008. There are trained and active HSAs providing services across all of Malawi's 29 districts. Overall utilization of HSA services in Malawi is higher than at the start of the program in 2009,^10^ and higher than in similar programs in other African countries.^11^ Although such progress is noteworthy, nationally, Malawi is not reaching the 80% benchmarks for supply and supervision indicators set by the MOH.

The survey shows that implementation strength and utilization of iCCM services varies widely at the district level (Table 5). We reviewed the results with national MOH program leadership and other partners supporting the implementation of iCCM to identify factors that might explain the district-level variability. Findings of particular importance to those implementing iCCM programs are discussed here.

Districts in which iCCM program activities were targeted to district-defined HTRAs had, on average, HSAs that were more active and seeing greater numbers of sick children than districts in which iCCM program activities were not targeted only to HTRAs. Five districts in the Southern Zone supported by the NGO partner Population Services International (Machinga, Neno, Mwanza, Thyolo, and Zomba) adopted a universal coverage strategy that aimed to train and deploy all HSAs for iCCM, regardless of whether they worked in an HTRA. Survey findings for training, deployment, and utilization indicators were consistent with differences in targeting strategy, with the five universal coverage districts among those showing the lowest rates of utilization per HSA. This finding suggests that, in non-HTRAs, children were most likely taken for care to health facilities rather than to the HSAs.

The survey findings suggest an association between HSAs living in their catchment area and increased utilization by the population. Among districts in the highest tertile for indicator 6 (HSA residence in the catchment area), only one was in the lowest tertile for the utilization indicator whereas among those in the lowest tertile for indicator 6, only one was in the highest tertile for the utilization indicator.

Given that both mentorship and restocking of HSAs supplies occur with supervisor assistance at the health facility, we predicted that the two indicators would perform similarly at district level. Unexpectedly, only two of the nine districts in the highest tertile for the drug and supplies indicator were also in the highest tertile for the supervision indicator (Kasungu, Blantyre), and three of these higher performing districts for drugs and supplies were in the lowest supervision tertile (Nkhata Bay, Rumphi, and Likoma).

Supervision appears to occur more frequently in districts with NGO partner support (Table 6). Six districts (Kasungu, Blantyre, Mulanje, Phalombe, Dowa, and Mchinji) achieved the MOH target of 80% or higher coverage of TWG indicator 5 (supervised in the last 3 months with reinforcement of clinical practice). Four of the highest-performing districts for this indicator (Blantyre, Mulanje, Dowa, and Mchinji) were partnered with the NGO STC for the majority of scale-up period. The other two (Kasungu, Phalombe) were partnered with WHO and UNICEF at the outset followed by Management Sciences for Health. In contrast, all five districts with no major NGO implementing partner support (Nkhata Bay, Rumphi, Likoma, Chitipa, and Salima) ranked in the bottom tertile for supervision.

Successful supply chain systems did not appear to depend on the support of large NGOs, as several districts without major NGO implementing partner support were among the top performers in this area (Nkhata Bay, Rumphi, and Likoma). Since 2011, the MOH has been rolling out a district-level supply chain system management tool called cStock (see Box 1). Among the nine top-performing districts for indicator 3a, five were supported by the new system (Mzimba North, Nkhata Bay, Kasungu, Nkhotakota, and Nsanje); all of these except Mzimba North had been using cStock since 2011 (i.e., early adopters) (Table 6). Five of the nine worst performing districts for indicator 3a were also implementing cStock although all had adopted the system more recently. Unexpectedly, Rumphi had neither a major NGO implementation partner nor cStock, but still reached the 80% MOH target for indicator 3a.

Districts with consistently higher scores demonstrate that MOH implementation strength targets are achievable with the right combination of supportive structures. Overall, Kasungu was the most consistently high-scoring district for the core implementation strength elements including proportion of iCCM-trained HSAs providing services as well as the supply and supervision related indicators. Kasungu also ranks in the highest tertile for proportion of HSAs reporting working in HTRA and proportion of HSAs residing in their catchment area. Utilization was also high in Kasungu. It is notable that Kasungu is perhaps a unique best-case scenario in terms of government and partner support. The district was one of the original rapid scale-up districts that received WHO and UNICEF support early in implementation, and a pilot district for cStock. Kasungu had stable district-level management and consistent support from other NGO partners since 2008. It was also one of two districts that participated in a U.S. Agency for International Development–funded pilot project focused on improving capture, reporting, and utilization of iCCM monitoring data by HSAs. The project included site visits to Kasungu and Dowa districts between February and May 2013.^12^ However, Nsanje, Phalombe, and Balaka had a similar set of factors (other than the pilot data project), but did not have same consistently high results across the implementation strength indicators. One possible explanatory factor is that compared with Kasungu, these three districts are located further from the capital city Lilongwe and potentially received less frequent oversight from the more senior central MOH and NGO staff.

The cell phone methodology worked well in Malawi. Malawi's cellular signal coverage rates are quite high, with more than 93% of areas covered.^13^ We were able to reach 99.7% of HSAs identified by the MOH. More than half of these HSAs were reached within a single call attempt, and 93% were reached within four call attempts.

There are limitations that might influence the interpretation and application of our findings. As designed, the study provides a snapshot of iCCM program implementation at a specific point in time. The indicators related to iCCM training and supply chains are particularly responsive to time-sensitive events, including training cycles (e.g., additional training could have taken place just weeks before or after the survey), and high-level supply chain issues (e.g., national-level stockouts). The cell phone interview approach was validated against supervisor reports and written supervision records, which themselves are subject to reporting bias. In the validation study, the sensitivity and specificity of the supply indicators were lower than others but all indicators were above the 80% threshold we set for adequacy.^3^ According to the national MOH, there were no system-wide interruptions in iCCM drug supplies during the time of the survey. The survey was subject to MOH-level limitations around identifying the official HTRA HSA catchment areas, and therefore not able to report on the official indicator 2b. The survey does not include household-level reports of service delivery, and it does not capture whether iCCM utilization by households is adequate compared with the expected burden of disease and access to health facilities. A large household survey was conducted by the NSO shortly after the census of HSAs, and includes some data that might help answer these questions. Despite these limitations, the cell phone methodology produced much-needed and consistently collected information on iCCM implementation strength across all districts, which was put to use immediately by the MOH and partners to improve program implementation. Work is under way now to compare the results of the implementation strength snapshot to those routine monitoring reports collected from implementing partners, to determine whether there are systematic biases in the routine systems that might be able to be addressed. The scale-up of the cStock system, which was in pilot phase during this study, to all districts in 2014 should improve the completeness of routinely reported supply indicators in Malawi. We recommend the telephone method for use in other contexts that lack strong routine or real-time monitoring systems, or as a tool to help periodically assess the quality and completeness of routinely collected data, although there may be limits in settings where cell phone coverage is less complete than in Malawi.

## Supplementary Material

Supplemental Table.

## Figures and Tables

**Table 1:** Overview of Malawi IMCI Technical Working Group consensus iCCM implementation strength indicator definitions

Element	Indicator	Numerator	Denominator
Active HSA	1. HSA-to-population ratio	HSAs working at time of assessment (Data source: *MOH district records*)	Total population under 5 years (Data source: *NSO Census 2008* - 2013 Projection)
Training	2a. Proportion of HSAs trained in iCCM	HSAs trained in iCCM	HSAs working at time of assessment (*MOH district records*)
Deployment	2b. *Proportion of hard-to-reach areas with iCCM-trained HSA*	*HSAs trained in iCCM who work in HTRA (*Data *not available)*	*Total no. of HTRA (*Data *not available)*
	2c. Proportion of iCCM HSAs who have seen a sick child in the past 7 days	HSAs who have seen a sick child in the past 7 days	Surveyed HSAs working in iCCM at the time of the assessment
	6. Proportion of iCCM HSAs who are living in their catchment area	HSAs who live in their catchment area	Surveyed HSAs working in iCCM at the time of the assessment
Drug supply and equipment	3a. Proportion of iCCM HSAs with supply of key iCCM drugs in last 3 months	HSAs with no stockouts of more than 7 days of co-trimoxizole, lumefantrine–artemether, ORS, and/or zinc in the last 3 months (HSA must have at least one dose of unexpired drug at the time of survey)	Surveyed HSAs working in iCCM at the time of the assessment
	3b. Proportion of iCCM HSAs with supply of life-saving CCM drugs in the last 3 months	HSAs with no stockouts of any duration of three life-saving drugs (co-trimoxizole, lumefantrine–artemether, and ORS) in the last 3 months (HSA must have at least one dose of unexpired drug at the time of survey)	Surveyed HSAs working in iCCM at the time of the assessment
Supervision	4. Proportion of iCCM HSAs supervised at village clinic in the last 3 months	HSAs supervised at village clinic in CCM in the last 3 months	Surveyed HSAs working in iCCM at the time of the assessment
	5. Proportion of iCCM HSAs supervised in the last 3 months with reinforcement of clinical practice	HSAs supervised at village clinic with observation of case management or practicing case scenarios or mentored in health facility in the last 3 months	Surveyed HSAs working in iCCM at the time of the assessment

HSAs = Health Surveillance Assistants; HTRA = hard-to-reach areas; iCCM = Integrated Community Case Management; IMCI = Integrated Management of Childhood Illness; MOH = Ministry of Health; NSO = National Statistical Office; ORS = oral rehydration solution.

**Table 2:** Implementation strength indicators by district: active HSAs and training

Zone	District	Total HSAs	Active HSAs	Training
			1. HSA-to-U5 population ratio (per 1,000)	2a. % Trained in iCCM
Northern	Chitipa	140	3.2	34.3
	Karonga	179	2.9	36.9
	Likoma	10	6.1	90.0
	Mzimba North	234	3.0	37.2
	Mzimba South	338	3.0	59.8
	Nkhata Bay	176	3.9	30.7
	Rumphi	153	4.2	26.8
Central	Dedza	460	3.5	22.8
	Dowa	428	3.0	58.4
	Kasungu	507	3.2	24.5
	Lilongwe	1,065	2.5	11.7
	Mchinji	343	3.0	40.2
	Nkhotakota	227	3.2	60.4
	Ntcheu	412	3.8	32.5
	Ntchisi	195	3.4	79.5
	Salima	303	3.9	22.4
Southern	Balaka	268	3.8	36.2
	Blantyre	600	2.9	16.0
	Chikwawa	290	3.1	24.8
	Chiradzulu	233	4.4	17.6
	Machinga	348	3.2	80.5
	Mangochi	553	3.1	10.3
	Mulanje	413	4.3	17.2
	Mwanza	74	3.8	93.2
	Neno	75	2.8	93.3
	Nsanje	145	2.8	46.9
	Phalombe	251	3.9	23.9
	Thyolo	518	5.0	76.6
	Zomba	617	4.5	88.7
Total		9,555	3.3	38.4

HSAs = Health Surveillance Assistants; iCCM = Integrated Community Case Management.

**Table 3:** Background characteristics of the interviewed HSAs currently working in iCCM by district

Zone	District	No. of HSAs working in iCCM	Gender (male)		Educational level						Age		Years working as a HSA*		Years working in iCCM†	
					Primary school		Form 2		Form 4 *Malawi* Senior Certificate or diploma							
		*n*	*n*	%	*n*	%	*n*	%	*n*	%	Mean	SD	Mean	SD	Mean	SD
Northern	Chitipa	46	36	78.3	1	2.2	23	50.0	22	47.8	37.3	6.5	11.2	5.9	3.0	0.9
	Karonga	65	59	90.8	1	1.5	28	43.1	36	55.4	37.5	7.4	11.1	6.4	3.6	0.9
	Likoma	6	4	66.7	0	0.0	0	0.0	6	100.0	35.8	3.2	6.2	1.6	3.3	0.7
	Mzimba North	85	68	80.0	4	4.7	32	37.6	49	57.6	37.2	7.9	10.2	6.4	2.5	1.2
	Mzimba South	197	152	77.2	4	2.0	75	38.1	118	59.9	36.6	7.6	9.7	6.5	2.0	1.5
	Nkhata Bay	54	50	92.6	1	1.9	16	29.6	37	68.5	37.5	7.8	9.9	5.3	3.5	1.0
	Rumphi	40	34	85.0	0	0.0	13	32.5	27	67.5	34.7	6.4	9.1	5.2	3.1	0.7
Central	Dedza	91	75	82.4	3	3.3	38	41.8	50	54.9	36.5	6.4	9.9	5.6	2.6	1.4
	Dowa	245	174	71.0	7	2.9	92	37.6	146	59.6	34.7	6.9	8.2	5.1	2.6	0.8
	Kasungu	124	102	82.3	1	0.8	54	43.5	69	55.6	36.0	6.9	8.1	4.7	3.7	1.1
	Lilongwe	127	112	88.2	2	1.6	49	38.6	76	59.8	37.5	7.5	10.3	6.0	3.1	1.2
	Mchinji	120	104	86.7	0	0.0	43	35.8	77	64.2	36.2	5.5	9.4	4.9	2.6	1.2
	Nkhotakota	135	82	60.7	1	0.7	66	48.9	68	50.4	35.6	6.8	9.4	5.8	2.6	0.8
	Ntcheu	129	98	76.0	5	3.9	51	39.5	73	56.6	36.5	7.4	9.5	5.9	2.9	1.2
	Ntchisi	97	75	77.3	2	2.1	42	43.3	53	54.6	35.6	6.5	8.9	5.1	1.8	1.4
	Salima	62	56	90.3	1	1.6	24	38.7	37	59.7	36.0	6.1	9.5	5.9	3.3	1.0
Southern	Balaka	96	74	77.1	3	3.1	41	42.7	52	54.2	36.0	6.1	9.4	5.6	3.4	1.2
	Blantyre	95	67	70.5	1	1.1	25	26.3	69	72.6	34.6	5.4	7.8	4.6	2.5	0.9
	Chikwawa	72	63	87.5	3	4.2	22	30.6	47	65.3	37.1	7.6	9.8	5.8	3.5	1.0
	Chiradzulu	40	33	82.5	0	0.0	16	40.0	24	60.0	34.1	5.5	7.3	4.0	3.8	0.9
	Machinga	262	165	63.0	19	7.3	121	46.2	122	46.6	36.8	6.9	10.8	5.7	2.1	1.0
	Mangochi	55	41	74.5	2	3.6	31	56.4	22	40.0	35.0	6.1	8.1	4.9	3.7	0.9
	Mulanje	71	57	80.3	2	2.8	29	40.8	40	56.3	36.2	6.1	8.8	5.4	3.3	0.7
	Mwanza	68	39	57.4	4	5.9	30	44.1	34	50.0	37.8	7.0	12.2	5.4	2.3	0.9
	Neno	67	40	59.7	1	1.5	22	32.8	44	65.7	38.6	7.8	12.7	7.3	2.6	0.9
	Nsanje	68	48	70.6	3	4.4	38	55.9	27	39.7	39.3	7.0	12.2	5.7	3.8	0.9
	Phalombe	58	46	79.3	2	3.4	19	32.8	37	63.8	35.2	6.2	9.0	5.1	3.6	1.0
	Thyolo	320	189	59.1	11	3.4	143	44.7	166	51.9	36.4	6.4	9.9	5.7	2.2	0.9
	Zomba	497	310	62.4	10	2.0	181	36.4	306	61.6	34.2	6.1	8.0	4.7	2.3	1.1
Total		3,392	2,453	72.3	94	2.8	1,364	40.2	1,934	57.0	36.0	6.8	9.4	5.6	2.6	1.2

HSAs = Health Surveillance Assistants; iCCM = Integrated Community Case Management.

**Table 4:** Implementation strength indicators by district: deployment, drugs and supplies, and supervision

Zone	District	No. of HSAs working in iCCM	Deployment			Drug supply and equipment		Supervision	
			% Self-report working in HTRA	2c. % Providing iCCM services	6. % Living in catchment area	3a. % With key drugs in the last 3 months	3b. % With life-saving medicines in the last 3 months	4. % Supervised in the last 3 months	5. Supervised w/ reinforcement of clinical practice in the last 3 months
Northern	Chitipa	46	97.8	80.4	97.8	67.4	63.0	21.7	21.7
	Karonga	65	93.8	80.0	69.2	58.5	43.1	32.3	52.3
	Likoma	6	100.0	83.3	16.7	100.0	100.0	33.3	33.3
	Mzimba North	85	83.5	94.1	96.5	85.9	58.8	56.5	64.7
	Mzimba South	197	93.9	87.3	78.7	67.5	53.3	31.5	60.4
	Nkhata Bay	54	96.3	92.6	75.9	81.5	55.6	37.0	42.6
	Rumphi	40	82.5	85.0	90.0	80.0	70.0	12.5	30.0
Central	Dedza	91	98.9	94.5	97.8	46.2	48.4	29.7	46.2
	Dowa	245	97.1	84.1	89.8	64.1	62.0	61.6	80.0
	Kasungu	124	96.8	93.5	91.9	75.0	54.0	94.4	96.0
	Lilongwe	127	98.4	89.8	56.7	44.9	40.2	51.2	69.3
	Mchinji	120	95.0	88.3	84.2	25.8	21.7	70.8	80.0
	Nkhotakota	135	81.5	93.3	77.0	77.0	74.1	53.3	66.7
	Ntcheu	129	95.3	93.0	93.8	60.5	55.0	22.5	45.7
	Ntchisi	97	96.9	84.5	92.8	59.8	51.5	56.7	78.4
	Salima	62	98.4	87.1	75.8	41.9	30.6	24.2	38.7
Southern	Balaka	96	89.6	80.2	56.3	49.0	39.6	65.6	67.7
	Blantyre	95	100.0	71.6	87.4	71.6	66.3	88.4	93.7
	Chikwawa	72	98.6	77.8	79.2	58.3	54.2	26.4	26.4
	Chiradzulu	40	100.0	85.0	75.0	45.0	40.0	17.5	37.5
	Machinga	262	66.4	57.3	27.5	58.4	54.6	64.9	71.4
	Mangochi	55	96.4	85.5	85.5	30.9	40.0	23.6	34.5
	Mulanje	71	94.4	81.7	60.6	52.1	25.4	67.6	83.1
	Mwanza	68	75.0	51.5	45.6	30.9	20.6	25.0	50.0
	Neno	67	88.1	58.2	52.2	79.1	82.1	29.9	53.7
	Nsanje	68	97.1	86.8	44.1	91.2	89.7	45.6	55.9
	Phalombe	58	100.0	87.9	94.8	48.3	50.0	65.5	82.8
	Thyolo	320	68.8	60.9	63.8	46.6	41.9	20.6	30.9
	Zomba	497	78.1	60.8	50.9	59.8	52.9	24.7	40.2
Total		3,392	87.1	77.0	69.5	58.8	51.6	43.7	57.6

HSAs = Health Surveillance Assistants; HTRA = hard-to-reach areas; iCCM = Integrated Community Case Management.

**Table 5:**
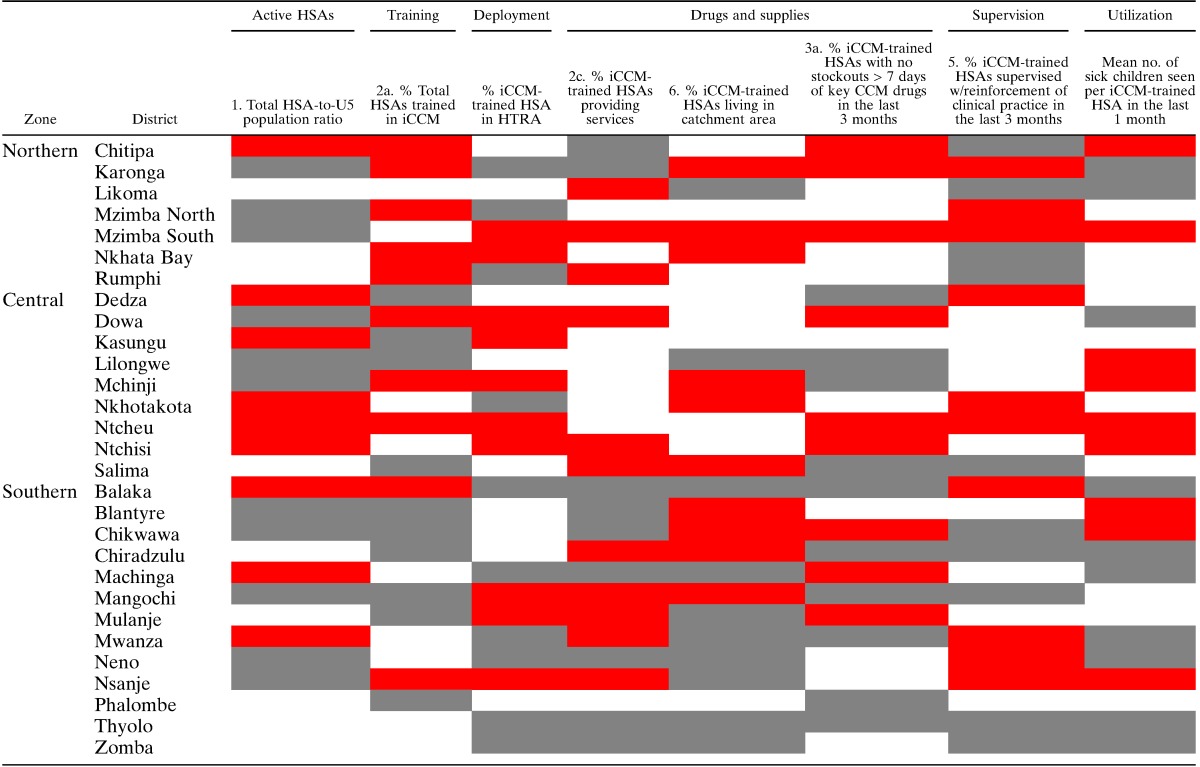
District ranking by tertile for select indicators of implementation strength and utilization

HSAs = Health Surveillance Assistants; HTRA = hard-to-reach areas; iCCM = Integrated Community Case Management.

White shading indicates that the district falls in the highest tertile for the indicator. Red shading indicates the middle tertile and gray shading indicates the lowest tertile.

**Table 6:** Core implementing partners by district at key stages in implementation

Zone	District	Original rapid scale-up district (2008)	Primary NGO implementation partners		JSI cStock (mid-2013)
			End 2009	Mid-2013	
Northern	Chitipa	No	–	SSDI	No
	Karonga	Yes	WHO/UNICEF	SSDI	Yes
	Likoma	No	–	–	No
	Mzimba North	Yes	WHO/UNICEF	–	Yes
	Mzimba South	Yes	WHO/UNICEF	–	Yes
	Nkhata Bay	No	–	–	Yes (2011)
	Rumphi	No	–	–	No
Central	Dedza	Yes	WHO/UNICEF	–	Yes
	Dowa	No	STC	SSDI	No
	Kasungu	Yes	WHO/UNICEF, MSH/Basics	SSDI	Yes (2011)
	Lilongwe	Yes	WHO/UNICEF	SSDI	Yes
	Mchinji	No	STC	–	No
	Nkhotakota	No	STC, MSH/Basics	SSDI	Yes (2011)
	Ntcheu	Yes	WHO/UNICEF	–	Yes
	Ntchisi	No	STC	–	Yes
	Salima	No	–	SSDI	No
Southern	Balaka	Yes	WHO/UNICEF, MSH/Basics	SSDI	Yes
	Blantyre	No	STC	–	No
	Chikwawa	No	MSH/Basics	SSDI	No
	Chiradzulu	Yes	WHO/UNICEF	–	Yes
	Machinga	No	PSI	SSDI	Yes (2011)
	Mangochi	No	MSH/Basics	SSDI	No
	Mulanje	No	STC	SSDI	Yes (2011)
	Mwanza	No	PSI	–	No
	Neno	No	PSI	–	No
	Nsanje	Yes	WHO/UNICEF, MSH/Basics	SSDI	Yes (2011)
	Phalombe	Yes	WHO/UNICEF, MSH/Basics	SSDI	Yes
	Thyolo	No	PSI	–	No
	Zomba	No	PSI, MSH/Basics	SSDI	No

JSI = John Snow Inc.; MSH = Management Sciences for Health; NGO = nongovernmental organizations; PSI = Population Services International; SSDI = Support for Service Delivery and Integration; STC = Save the Children; WHO/UNICEF = World Health Organization/ United Nations Children's Fund.
