# Supplementary material for: Measuring Implementation Strength for Integrated Community Case Management in Malawi: Results from a National Cell Phone Census
Source: Am J Trop Med Hyg. 2015 Oct 7;93(4):861–8. doi: 10.4269/ajtmh.14-0797 (PMC4596612; doi:10.4269/ajtmh.14-0797)
Supplement: Supplementary file 1 [file SD9.pdf]

Supplemental Table 1

Mean number of sick children seen in previous 7 days by HSAs providing iCCM services

		HSAs working in iCCM	2c. % Providing iCCM services (saw sick child in last 7 days)		No. of sick children treated in previous week[1]	
Zone	District	*n*	*n*	%	Mean	SD
Northern	Chitipa	46	37	80.4	15.9	16.1
	Karonga	65	52	80.0	12.1	13.6
	Likoma	6	5	83.3	3.4	2.3
	Mzimba North	85	80	94.1	14.5	10.7
	Mzimba South	197	172	87.3	15.9	12.8
	Nkhata Bay	54	50	92.6	22.3	17.2
	Rumphi	40	34	85.0	26.2	24.3
Central	Dedza	91	86	94.5	17.6	12.8
	Dowa	245	206	84.1	10.8	7.8
	Kasungu	124	116	93.5	21.6	14.6
	Lilongwe	127	114	89.8	20.9	19.5
	Mchinji	120	106	88.3	16.7	11.7
	Nkhotakota	135	126	93.3	22.3	19.8
	Ntcheu	129	120	93.0	11.4	8.1
	Ntchisi	97	82	84.5	14.4	9.2
	Salima	62	54	87.1	20.1	16.6
Southern	Balaka	96	77	80.2	13.6	8.3
	Blantyre	95	68	71.6	16.7	11.3
	Chikwawa	72	56	77.8	15.9	13.0
	Chiradzulu	40	34	85.0	13.4	9.1
	Machinga	262	150	57.3	12.9	8.8
	Mangochi	55	47	85.5	25.6	31.2
	Mulanje	71	58	81.7	15.9	9.3
	Mwanza	68	35	51.5	8.2	5.8
	Neno	67	39	58.2	13.2	11.4
	Nsanje	68	59	86.8	16.4	11.9
	Phalombe	58	51	87.9	19.6	15.0
	Thyolo	320	195	60.9	8.4	7.7
	Zomba	497	302	60.8	11.1	10.1
TOTAL		3,392	2,611	77.0	15.1	13.6

HSAs = Health Surveillance Assistants; iCCM = Integrated Community Case Management; SD = standard deviation.
